# A clinical study to evaluate meibomian gland dysfunction and tear film changes in patients with pterygium

**DOI:** 10.6026/973206300214504

**Published:** 2025-12-15

**Authors:** Neda Rahman, Sunil Kumar, Rohit Kumar Mahato, Deepankar Deepankar, Mousmi Anand

**Affiliations:** 1 Regional Institute of Ophthalmology, Rajendra Institute of Medical Sciences, Ranchi, Jharkhand, India

**Keywords:** Pterygium, meibomian gland dysfunction, tear film, dry eye, ocular surface disease, tear film break-up time, schirmer's test

## Abstract

Pterygium is a degenerative ocular surface disorder whose relationship with meibomian gland dysfunction (MGD) and tear film instability remains understudied, especially in Eastern India. This case-control study included 110 adults to evaluate tear film parameters and MGD status in pterygium patients compared with healthy controls. Pterygium cases showed significantly higher rates of Schirmer's I, TBUT and TMH abnormalities, with strong correlations between tear instability and MGD severity. Higher pterygium grades demonstrated the most marked tear film disruption and meibomian gland changes. Thus, we show the need for early detection and management of MGD and dry eye to improve outcomes in pterygium patients.

## Background:

Pterygium represents a degenerative fibrovascular disease of the ocular surface that poses significant challenges to both patient comfort and visual function. This condition manifests as abnormal conjunctival tissue growth extending onto the cornea, creating a wing-like appearance that can cause substantial discomfort, corneal abnormalities and aesthetic concerns [1]. The global prevalence of pterygium ranges from 10.2% to 12%, with notably higher rates observed in tropical and subtropical regions where environmental factors such as ultraviolet radiation exposure, dust and wind contribute to its development [2]. The pathophysiology of pterygium involves a complex interplay of genetic and environmental factors. Geographic latitude, rural living conditions, advanced age, race, gender, chronic sunlight exposure and persistent irritation and inflammation have all been identified as significant risk factors [3]. The condition demonstrates a clear association with outdoor occupations and activities that increase exposure to ultraviolet radiation, suggesting that reactive oxygen species generated by UV exposure may play a crucial role in pterygium formation [4]. Numerous clinical studies have established a connection between pterygium and alterations in the ocular surface environment, particularly regarding tear film stability and dry eye syndrome. According to the International Dry Eye Workshop, dry eye is defined as a multifactorial disease affecting the ocular surface and tear film, characterized by symptoms of discomfort, visual disruption, tear film instability and potential ocular surface damage. The condition is associated with increased tear film osmolarity and inflammation of the ocular surface [5]. The tear film consists of two primary components: an aqueous layer produced by the lacrimal glands and a lipid layer containing fatty components secreted by the meibomian glands. Meibomian glands are specialized sebaceous glands located within the eyelids that secrete lipid-rich meibum into the tear film to prevent excessive tear evaporation [6]. These glands play a crucial role in maintaining tear film stability and preventing evaporative dry eye. Meibomian gland dysfunction (MGD) represents a chronic, generalized disorder of the meibomian glands, typically characterized by terminal duct obstruction and qualitative or quantitative alterations in glandular secretion. This condition can lead to tear film changes, ocular surface disease, clinically significant inflammation and symptoms of eye discomfort [7, 8- 9]. MGD is often overlooked in clinical practice but may represent the missing link between pterygium and dry eye syndrome. Previous research has demonstrated that pterygium causes pathological alterations to the conjunctiva, cornea and eyelids, resulting in disrupted tear film function. Despite the established relationship between pterygium and dry eye, the specific role of meibomian gland dysfunction in this association remains inadequately characterized. The inflammatory nature of pterygium, with its marked inflammatory infiltrate and excessive cytokine production, may contribute to changes in meibomian gland structure and function. Elevated levels of inflammatory factors such as TNF-α, IL-4 and IL-5 can damage lid margins and lead to alterations in meibomian glands [10]. Therefore, it is of interest to evaluate the relationship between pterygium and meibomian gland dysfunction, assess tear film changes in pterygium patients and determine the correlation between pterygium severity and the degree of MGD and dry eye symptoms.

## Materials and Methods:

##  Study design:

This investigation was conducted as a hospital-based case-control observational study at the Regional Institute of Ophthalmology, Rajendra Institute of Medical Sciences, Ranchi. The study design allowed for comprehensive comparison between patients with pterygium and matched controls without ocular surface abnormalities.

## Study population:

The study population consisted of patients attending the outpatient department of the Department of Ophthalmology at the Regional Institute of Ophthalmology from July 2023 to December 2024. A total of 110 patients were enrolled in the study, with 55 patients in the case group (pterygium patients) and 55 patients in the control group (normal eyes matched for age and sex).

## Inclusion and exclusion criteria:

Inclusion criteria for the case group included patients diagnosed with pterygium in one or both eyes between the ages of 21 and 60 years. Control group participants were individuals with normal eyes matched for age and sex without any ocular surface abnormalities. Exclusion criteria were established to eliminate confounding factors that could affect tear film stability or meibomian gland function. These included individuals with corneal conditions or scarring, contact lens use within the previous three months, cicatricial ocular surface disorders, eye allergies, history of ocular injury or surgical procedures, infectious diseases of the cornea or conjunctiva and systemic conditions affecting tear or lipid production such as Sjögren's disease, diabetes, dyslipidemia and acne rosacea.

## Methodology:

A comprehensive medical history was obtained from each participant, including demographic information, occupational details, presenting symptoms, disease duration, progression patterns and associated conditions related to pterygium. Complete ocular examinations were performed, including visual acuity assessment using Snellen's chart and detailed slit-lamp examination. Pterygium evaluation included staging differentiation between resting and progressive forms. Resting stage pterygium was identified by thin, flat tissue with minimal redness and no spontaneous regression. Progressive stage pterygium was characterized by larger, thicker tissue displaying hypertrophy and increased redness with numerous blood vessels, obscured underlying sclera and potential extension into the corneal pupillary area. Pterygium grading was performed using a standardized three-grade system. Grade 1 pterygium was defined as pterygial tissue reaching the limbus. Grade 2 pterygium extended to a point midway between the limbus and pupillary margin. Grade 3 pterygium extended beyond the pupillary margin. Tear film parameter evaluation included three standardized tests. The Schirmer's I test was performed using sterile strips measuring 5x35mm positioned between the lateral one-third and medial two-thirds of the lower eyelid in the fornix. The wetted strip length was measured after five minutes, with values less than 10mm indicating tear film instability. Tear Film Break-up Time (TBUT) was assessed using fluorescein strips moistened with preservative-free normal saline, placed on the inferior fornix with the patient instructed to blink once. The tear film was observed under slit-lamp with cobalt blue filter and the time between the last blink and appearance of the first dry spot was recorded as the average of three attempts. TBUT values under 10 seconds indicated tear film instability. Tear Meniscus Height (TMH) was measured using fluorescein strips moistened with normal saline placed on the lower eyelid with the patient looking upward. The tear meniscus was observed using cobalt blue filter under slit-lamp and the tear film height was measured. TMH values under 0.3mm indicated tear film instability. Meibomian gland dysfunction evaluation included assessment of lid margin abnormalities, gland expressibility and meibum quality. Lid margin abnormalities were scored from 0-4 based on the presence of dilated blood vessels, blocked meibomian gland openings, mucocutaneous junction displacement, or eyelid margin irregularities. Meibomian gland expressibility was evaluated using diffuse light under slit-lamp, with five central meibomian glands assessed and expressed. Scoring ranged from 0-3, with 0 indicating secretion in all five glands, 1 indicating secretion in three to five glands, 2 indicating secretion in one to two glands and 3 indicating absent secretion in all glands. Total scores for individual eyes including both upper and lower eyelids ranged from 0-6. Meibum quality scoring assessed lipid secretion characteristics. Score 0 indicated clear or light yellow secretion, score 1 indicated creamy yellow secretion, score 2 indicated granular white or yellow secretion and score 3 indicated toothpaste-like consistency. Total scores ranged from 0-6.

## Statistical analysis:

Descriptive statistics were used to represent baseline and demographic characteristics of study participants. Categorical data were presented as numbers and percentages, while continuous data were expressed as means and standard deviations for normally distributed variables. Chi-square tests were performed to determine differences in categorical data between groups. Spearman's correlation coefficients were calculated to assess associations between tear film parameters and meibomian gland dysfunction scores. Statistical analysis was completed using SPSS software, STATA/MP 18.5 and Jamovi. P-values less than 0.05 were considered statistically significant.

## Ethical considerations:

Ethical approval was obtained from the Institutional Ethics Committee of Rajendra Institute of Medical Sciences with memo number 100, dated March 9, 2024. The entire study was conducted in accordance with the Declaration of Helsinki principles. Written informed consent was obtained from all participants prior to enrollment and examination.

## Results:

Age distribution analysis revealed that pterygium predominantly affected middle-aged individuals. In the pterygium group, 4 patients (7.27%) were aged 21-30 years, 14 patients (25.45%) fell within the 31-40 years range, 26 patients (47.27%) were in the 41-50 years age group and 11 patients (20%) belonged to the 51-60 years category. The control group showed similar age distribution with 6 patients (10.9%) in the 21-30 years group, 16 patients (29.09%) in the 31-40 years group, 22 patients (40%) in the 41-50 years group and 11 patients (20%) in the 51-60 years group, as shown in Table 1 (see PDF). Gender distribution analysis demonstrated male predominance in both groups. Among the 55 pterygium patients, 31 (56.36%) were males and 24 (43.64%) were females. In the control group, 35 (63.63%) were males and 20 (36.36%) were females, indicating comparable gender distribution between the two groups. Side distribution analysis in the pterygium group revealed that 27 patients (49.09%) had pterygium affecting the right eye alone, 10 patients (18.18%) had left eye involvement only and 18 patients (32.72%) presented with bilateral pterygium. This distribution resulted in a total of 73 affected eyes for detailed analysis. Pterygium staging classification showed that 51 eyes (69.87%) were in the resting stage, characterized by thin, flat tissue with minimal redness, while 22 eyes (30.13%) were in the progressive stage, demonstrating larger, thicker tissue with increased vascularity and inflammatory signs. Location analysis demonstrated that nasal pterygium was significantly more common than temporal pterygium. Among the 73 affected eyes, 63 eyes (86.30%) had nasal pterygium while only 10 eyes (13.70%) had temporal pterygium, consistent with established patterns of pterygium distribution. Pterygium grading assessment revealed that Grade 1 pterygium was most prevalent, affecting 37 eyes (50.7%), followed by Grade 2 pterygium in 21 eyes (28.8%) and Grade 3 pterygium in 15 eyes (20.5%). This distribution is presented in Table 2 (see PDF).

Schirmer's I test results demonstrated significant differences between pterygium patients and controls. Among the 73 pterygium eyes, 20 eyes (27.4%) showed abnormal values less than 10mm, while 53 eyes (72.6%) had normal measurements greater than 10mm. In contrast, only 2 eyes (2.7%) in the control group showed abnormal values, while 71 eyes (97.3%) demonstrated normal measurements. The difference was statistically significant (p=0.0035), indicating substantially reduced tear production in pterygium patients. Tear Film Break-up Time (TBUT) analysis revealed even more pronounced differences between groups. In the pterygium group, 28 eyes (38.36%) demonstrated abnormal TBUT values less than 10 seconds, while 45 eyes (61.64%) showed normal values greater than 10 seconds. The control group showed only 3 eyes (4.1%) with abnormal values and 70 eyes (95.9%) with normal values. This difference was highly statistically significant (p<0.00001), indicating severe tear film instability in pterygium patients. Tear Meniscus Height (TMH) assessment showed that 37 pterygium eyes (50.7%) had abnormal values less than 0.3mm, while 36 eyes (49.3%) maintained normal values greater than 0.3mm. In the control group, only 10 eyes (13.7%) showed abnormal values, while 63 eyes (86.3%) demonstrated normal measurements. This difference was highly statistically significant (p<0.00001), as demonstrated in Table 3 (see PDF). Comparative analysis of meibomian gland parameters between pterygium and control groups revealed important differences. The mean lid abnormalities score was 0.616 in the pterygium group compared to 0.274 in controls, though this difference was not statistically significant (p=0.336). The mean meibomian gland expressibility score was 0.438 in pterygium patients versus 0.247 in controls, showing borderline significance (p=0.033). The mean meibum score was 0.534 in pterygium patients compared to 0.425 in controls, but this difference was not statistically significant (p=0.307). Spearman's correlation analysis revealed significant associations between tear film parameters and meibomian gland dysfunction scores in pterygium patients. Schirmer's, I test showed significant negative correlations with lid abnormalities (ρ=-0.507, p<0.001), meibomian gland expressibility (ρ=-0.394, p<0.001) and meibum values (ρ=-0.405, p<0.001). TBUT demonstrated significant negative correlations with lid abnormalities (ρ=-0.436, p<0.001), expressibility (ρ=-0.597, p<0.001) and meibum values (ρ=-0.559, p<0.001). TMH showed significant negative correlations with lid abnormalities (ρ=-0.680, p<0.001), expressibility (ρ=-0.453, p<0.001) and meibum values (ρ=-0.433, p<0.001). In the control group, correlations were less consistent. Schirmer's, I showed significant correlations with lid abnormalities (ρ=-0.599, p<0.001) and expressibility (ρ=-0.249, p=0.008), but not with meibum values. TBUT correlated significantly only with lid abnormalities (ρ=-0.489, p<0.001), while TMH showed significant correlations with lid abnormalities (ρ=-0.806, p<0.001) and expressibility (ρ=-0.420, p<0.001), but not with meibum values, as presented in Table 4 (see PDF). Analysis of the relationship between pterygium severity and clinical parameters revealed progressive deterioration with increasing grade. Mean Schirmer's I values decreased from 14.1mm in Grade 1 to 12.6mm in Grade 2 and 10.7mm in Grade 3 pterygium. Similarly, mean TBUT values decreased from 12.3 seconds in Grade 1 to 10.2 seconds in Grade 2 and 9.27 seconds in Grade 3. Mean TMH values also decreased from 0.238mm in Grade 1 to 0.229mm in Grade 2 and 0.133mm in Grade 3. Meibomian gland dysfunction parameters showed corresponding increases with pterygium severity. Mean lid abnormality scores increased from 0.432 in Grade 1 to 0.524 in Grade 2 and 1.20 in Grade 3. Mean expressibility scores increased from 0.00 in Grade 1 to 0.619 in Grade 2 and 1.27 in Grade 3. Mean meibum scores increased from 0.0270 in Grade 1 to 0.762 in Grade 2 and 1.47 in Grade 3, demonstrating progressive meibomian gland dysfunction with increasing pterygium severity, as shown in Table 5 (see PDF).

## Discussion:

This comprehensive study provides important insights into the relationship between pterygium and ocular surface changes, particularly focusing on tear film stability and meibomian gland dysfunction. The findings demonstrate that pterygium significantly affects tear film parameters and correlates with meibomian gland dysfunction, supporting the hypothesis that MGD may serve as a crucial link between pterygium and dry eye syndrome. The demographic characteristics observed in this study align with established patterns of pterygium prevalence. The predominance of pterygium in the 41-50 year age group (47.27%) is consistent with previous studies by Cameron and Youngson, who found pterygium to be more commonly observed in individuals above 40 years of age [11, 12]. The male predominance observed in this study (56.36% males versus 43.64% females) is consistent with several previous investigations, though some studies have reported higher prevalence in females [13]. The male predominance may be attributed to increased outdoor occupational exposure, as men are more frequently engaged in outdoor work compared to women, leading to greater ultraviolet light exposure [14]. This occupational association supports the established link between pterygium and environmental factors, particularly UV radiation exposure. The predominance of nasal pterygium (86.30%) over temporal pterygium (13.70%) observed in this study is consistent with established patterns reported in the literature [15, 16, 17, 18-19]. The preferential nasal location may be explained by the hypothesis that light reflection from the temporal orbital margin or nose onto the nasal conjunctiva contributes to limbal stem cell activation, potentially triggering pterygium formation. This pattern occurs despite equal sunlight exposure to both eyes during outdoor activities, suggesting that local anatomical factors may play a role in pterygium development. The pathophysiology underlying these tear film abnormalities in pterygium patients appears to be multifactorial. UV-induced genetic alterations may affect the production of inflammatory cytokines such as IL-6 and IL-8 in pterygium eyes. These cytokines can trigger the formation of matrix metalloproteinases (MMPs), which damage the ocular surface and cause tear film instability [20]. This leads to goblet cell loss, decreased mucus production and tear film hyperosmolarity, all contributing to dry eye syndrome development. The meibomian gland dysfunction assessment revealed interesting patterns, though the differences between groups were less pronounced than the tear film parameters. While the mean scores for lid abnormalities and meibum quality were not statistically significant between groups, the meibomian gland expressibility score showed borderline significance (p=0.033). This finding suggests that while MGD may be present in pterygium patients, it may be more subtle in its manifestation compared to the tear film abnormalities. The correlation analysis provided crucial insights into the relationship between tear film parameters and meibomian gland dysfunction in pterygium patients. All tear film parameters showed significant negative correlations with all meibomian gland dysfunction scores in the pterygium group, suggesting that as MGD severity increases, tear film stability decreases. These correlations were stronger and more consistent in the pterygium group compared to controls, suggesting that pterygium may amplify the relationship between MGD and tear film instability. The analysis of pterygium severity and clinical parameters revealed a clear progressive pattern. Grade 3 pterygium showed the most severe tear film abnormalities and highest meibomian gland dysfunction scores, suggesting that pterygium progression is associated with worsening ocular surface disease. This finding has important clinical implications, as it suggests that early intervention may be beneficial in preventing severe dry eye complications. The inflammatory nature of pterygium appears to play a crucial role in the development of MGD. Pterygium is characterized by marked inflammatory infiltrate with excessive cytokine production. Elevated levels of inflammatory factors such as TNF-α, IL-4 and IL-5 can damage lid margins and lead to alterations in meibomian glands [21]. Additionally, pterygium is associated with abnormalities in the limbal microenvironment, causing increased epithelial cell proliferation and keratinization of the lid margin and meibomian glands [22]. Pterygium is associated with significant alterations in tear film stability and meibomian gland function, with patients exhibiting higher Ocular Surface Disease Index scores, reduced tear break-up time, and increased meibomian gland dysfunction compared with healthy controls, indicating compromised ocular surface homeostasis in affected eyes [23]. Clinical evaluation revealed that a substantial proportion of pterygium patients show abnormalities on Schirmer's and TBUT testing along with varying grades of meibomian gland disorder, suggesting that tear film dysfunction may contribute to ocular discomfort and dry eye symptoms in this population [24]. Comparative studies further indicate that tear film dysfunction and meibomian gland abnormalities are more prevalent in eyes with pterygium than in non-pterygium eyes, emphasizing the importance of comprehensive tear film and eyelid margin assessment in the management of these patients [25]. The clinical implications of these findings are significant. The demonstration of tear film abnormalities and meibomian gland dysfunction in pterygium patients suggests that comprehensive ocular surface evaluation should be part of routine pterygium management. Early identification and treatment of MGD and dry eye in pterygium patients may improve overall patient comfort and potentially influence pterygium progression.

## Conclusion:

Pterygium is strongly associated with significant tear film instability and meibomian gland dysfunction, indicating broader ocular surface involvement rather than an isolated conjunctival disorder. The severity of these abnormalities increases with advancing pterygium grade, underscoring the progressive nature of the disease. Early evaluation and management of dry eye and MGD are essential to improve patient comfort and potentially slow disease progression.

## References

[1] Devebacak A (2023). Optom Vis Sci..

[2] Prabhasawat P (2020). Cornea..

[3] Dogan L (2023). Ocul Immunol Inflamm..

[4] Sun M (2024). Curr Eye Res..

[5] Mathur R (2023). Oman J Ophthalmol..

[6] Altin Ekin M (2020). Eye Contact Lens..

[7] Kim KY (2021). BMC Ophthalmol..

[8] Kim SE (2020). BMC Ophthalmol..

[9] Fatima A (2022). Exp Eye Res..

[10] Kiyat P (2020). Cornea..

[11] Lin X (2022). BMC Ophthalmol..

[12] Kim WJ (2022). Ann Med..

[13] Chen X (2024). Eye Contact Lens..

[14] Agin A (2020). Graefes Arch Clin Exp Ophthalmol..

[15] Liu T (2022). Front Med (Lausanne)..

[16] Gu T (2020). BMC Ophthalmol..

[17] Chang J (2024). Optom Vis Sci..

[18] Arita R (2024). PLoS One..

[19] Shen J (2024). Cornea..

[20] Shimizu S (2021). Cornea..

[21] Pan CY (2024). Int J Ophthalmol..

[22] García-Marqués JV (2021). Curr Eye Res..

[23] Sharma S (2021). Panacea J Med Sci..

[24] Kurahatti PK (2024). J Evol Med Dent Sci..

[25] Sharma N (2025). J Adv Med Pharm..

